# Intratumoral electroporation of plasmid interleukin-12: efficacy and biomarker analyses from a phase 2 study in melanoma (OMS-I100)

**DOI:** 10.1186/1479-5876-13-S1-O11

**Published:** 2015-01-15

**Authors:** Adil Daud, Alain Algazi, Michelle Ashworth, Michael Buljan, Kathryn Toshimi Takamura, Tu Diep, Robert H Pierce, Shailender Bhatia

**Affiliations:** 1 University of California, San Francisco – San Francisco, California, USA; 2 OncoSec Medical Inc. – San Diego, California, USA; 3 University of Washington – Seattle, Washington, USA

## Background

Recent data from immune checkpoint studies, including studies of anti-PD1 and anti-PDL1 antibodies, suggest that an inflammatory intratumoral milieu is required for an optimal response to immune therapy. Serial biopsy analyses from a phase 1 study demonstrate that transformation of tumor cells with electroporation (EP) of plasmid interleukin-12 (pIL-12) promotes this inflammatory immune milieu. Here we present clinical response data for 30 advanced melanoma patients (pts) treated with pIL-12 EP in a phase 2 trial (OMS-I100). We also present additional safety and more detailed biomarker data demonstrating the promotion of pro-inflammatory genes with pIL-12 EP therapy.

## Methods

Thirty patients with stage IIIB-IV melanoma received up to 4 cycles of pIL-12 EP into superficial cutaneous, subcutaneous, and nodal lesions on days 1, 5 and 8 of each 12-week cycle. Tumor responses were evaluated using modified RECIST criteria for cutaneous lesions. Adverse events (AEs) were assessed using CTCAE version 4. Alterations in transcription were assessed by comparing pre- and post-treatment biopsies from treated lesions using NanostringTM technology to identify pharmacodynamic markers of downstream pathway activation and to characterize cellular infiltration.

## Results

The best overall response rate (BORR) by modified RECIST in 29 evaluable pts was 31% (9/29), with 10% (3/29) of pts achieving a CR. Regression of at least one non-treated lesion was seen in 54% (13/24) of pts with evaluable lesions. The most common treatment-related adverse event (AE) reported was transient Grade 1/2 pain at the treatment site, reported in 87% (26/30) of pts. Grade 3 adverse events were rare and included only 1 report of Grade 3 pain at the injection site. No grade 4 or higher adverse events were observed. Analysis of tissue samples from patients treated with pIL-12 EP showed a gene expression pattern consistent with downstream activation of NK cells and interferon-γ-dependent genes, including key genes responsible for tumor inflammation, antigen processing and presentation (APM).

## Conclusions

pIL-12 EP monotherapy induces objective tumor responses in a significant proportion of patients (BORR 31%) and treatment was well tolerated. pIL-12 EP promotes the expression of pro-inflammatory genes including genes required for antigen processing and presentation. Regression of non-treated lesions suggests successful induction of systemic anti-tumor immune-mediated effects. Based on these data, further investigation of pIL-12 EP both as a single agent, and in combination with other therapies such as anti-PD1/PD-L1, is warranted.

